# Perspective and Experience of Male Nursing Students in 3-year Vocational College During Their Clinical Practicum: A Qualitative Study in Shanghai, China

**DOI:** 10.3389/fpubh.2022.905200

**Published:** 2022-05-30

**Authors:** Yue Liu, Chun Yao, Sijia Zhao, Peng Han, Jinxia Jiang, Xia Duan

**Affiliations:** ^1^Emergency Department, Shanghai Tenth People's Hospital, School of Medicine, Tongji University, Shanghai, China; ^2^Department of Nursing, Shanghai Jiguang Polytechnic College, Shanghai, China; ^3^Nursing Department, Shanghai First Maternity and Infant Hospital, School of Medicine, Tongji University, Shanghai, China

**Keywords:** faculty, male nursing student, preceptorship, qualitative study, education

## Abstract

**Background:**

With the expansion of nursing enrollment, more and more male students are joining the nursing program. Nevertheless, the number of male nurses who actually stay in clinical work in China continues to be low. Clinical practicum is a critical period during which nursing students transform to the role of registered nurses. However, few studies have researched the perspective and experience during clinical practicum among male nursing students in 3-year vocational colleges in China.

**Objectives:**

To explore the perspective and experience of Chinese male nursing students during clinical practicum in the third year. One of the main objectives is to provide implications for future nursing education and to help male nursing students for better adaptation to clinical roles.

**Methods:**

The qualitative study used a purposive sampling method and collected data through in-depth, semi-structured interviews. The participants were 20 male students from a 3-year vocational college in Shanghai. They completed the basic nursing course in college and underwent a clinical practicum in the 3rd level hospitals in China between July 2020 and March 2021. A Seven-stage Colaizzi process was used for data analysis.

**Results:**

Three themes were revealed through content analysis: opportunities and challenges brought by gender factors in hospital humanistic environment, gaining experience and growth during clinical practicum, and future career planning.

**Conclusions:**

The presented findings further our understanding of the perspective and experience of male nursing students during clinical practicum. It is very important and necessary for providing implications for future nursing education. At the same time, effective support from society, such as social unions, mass media, government, can help better address male nursing students' needs and complete the role transform of registered nurses.

## Introduction

Nursing is the most important part of the professional health care system. In fact, in 2020, there were about 28 million newly employed nurses and midwives. However, there is still a shortage of 5.9 million nurses worldwide (1). The shortage of human nursing resources is a global health system problem. It is well known that the nursing workforce has been consistently dominated by female workers (2). Support against educational stereotypes and role recognition have been especially important in encouraging men to opt for nursing careers (3). According to the WHO (2020), men make only about 10% of the world's nursing staff. In North American countries, 5.8 percent of nurses are male, compared with 10 percent in the UK and 4.9 percent in Japan (4).

Compared with developed countries, the proportion of men in the nursing professions in China is much smaller (5). In 2019, male nurses made up just 2.3 percent of the 4.09 million registered nurses (6). In addition, aging population in China is rapidly increasing. In 2021, the number of Chinese people over 65 years old soared to 190 million, or 13.5% of the population. To improve the demographic balance, the Chinese central government started encouraging couples to have three children in 2021. With many women of high maternal age rushing to have a third child, the incidence of adverse pregnancy outcomes such as fetal abnormalities and premature delivery will increase correspondingly, which brings difficult challenges to nursing work (7). It is obvious that the aging population and three children policy in China have increased the workload of nurses and further exposed the shortage of human nursing resources. Consequently, there is a definite need to increase the number of male nurses so as to alleviate the nursing workforce shortages. In addition, male nurses are believed to have certain physical advantages and be more stress-resilient in disastrous circumstances and some special departments (8). Considering aforementioned reasons, there is a steady and consistent demand for male nurses (9).

During the practicum phase, male nursing students are in the transition period between students and nurses. China began to recruit male nursing students in 1977 when the whole country restored the college entrance examination system (10). With the development of society and the need for professional development in nursing, hospitals have started paying more attention to male nurses because of their unique advantages. As a result, the number of male nursing students in various colleges and universities has significantly increased. According to enrollment data from a 3-year vocation college in Shanghai, male students accounted for 12% of the nursing students in 2016. Five years later, male students made up 32% of the nursing student population in 2021. Although recruitment of male nursing students has expanded over recent years, there were no significant breakthroughs in the number of men who eventually become registered nurses. As things stand, diversity of social work or non-clinical leadership positions may be more attractive to male nurses (11). The male nurse turnover intention rate is higher, with more than 83.3 percent and the proportion of clinical male nurses remains not high (12).

In China, nursing students attending 3-year vocational colleges stay in school for 2 years, which is less than undergraduate students. The vocational college courses are arranged as basic public courses (English, Computer, Physical Education, etc.), specialized basic courses (Physiology, Anatomy, Pharmacology, Biochemistry, Nosology, etc.), and specialized courses (Basic Nursing, Surgical Nursing, Medical Nursing, Nursing Psychology, Emergency, and Intensive Nursing, Obstetric and Gynecological Nursing, etc.). During the first 2 years, nursing students must complete all courses and obtain 126.5 credits. In the third year, they complete a clinical practicum in different teaching hospitals. Students began to fill in the application form of intention to attend clinical practicum in hospitals according to their own needs in March of the sophomore year, and most of them usually opt for 3^rd^ Level Hospital. The college assigns practicum hospitals according to the students' performance ranking. In the present study, 341 students entered 3^rd^ Level Hospital for practicum in July of the sophomore year, accounting for 65% of the students in the whole grade. The other students were admitted to the 2^nd^ Level Hospital for practicum. All practicum hospitals must meet the requirements of grade II or above and have teaching qualifications. Clinical practicum, which lasts for 36 weeks, i.e., from July of sophomore year to March of junior year, constitutes 1/3 of the total class hours of 3-year vocational nursing education in China. Educators make the practicum syllabus (Table 1). During the clinical practicum, students accept the dual leadership of the college and the nursing department of the practicum hospital. At the end of the practicum, students undergo comprehensive evaluation in combination with the graduation examination. Through the comprehensive clinical practicum, nursing students acquire basic nursing skills, such as intravenous infusion, intramuscular injection, urethral catheterization, and rescuing coordination. Nursing students are familiar with nursing evaluation, nursing measures and nursing operation of clinical diseases and with various medical instruments. At the same time, nursing students learn to communicate effectively with patients and medical workers. The clinical environment where students accomplish clinical practicum allows students to acquire the nursing ability and clinical skills and develop as professional nurses. In China's medical system, the clinical practicum, which has unique advantages, has an important role in nursing education. The practicum-based ability helps students to better cope with their work (13). There is growing evidence that clinical practicum is the foundation of nursing education that contributes to nursing skills acquisition and sustainability (14).

**Table 1:** Internship arrangement.

**Practicum**	**Internship provider**	**Internship time**	**Internship period (weeks)**
Internal medicine ward	3rd level hospital /2nd level hospital	2020.7–9	10 weeks
Surgical ward	3rd level hospital /2nd level hospital	2020.10–11	10 weeks
Obstetrics and gynecology department	3rd level hospital /2nd level hospital	2020.12	4 weeks
Pediatric ward	3rd level hospital /2nd level hospital	2020.1	4 weeks
Outpatient and emergency department	3rd level hospital /2nd level hospital	2021.2	3 weeks
Spring festival	/	2021.2	1 week
Operating room	3rd level hospital /2nd level hospital	2021.3	2 weeks
Intensive care unit	3rd level hospital /2nd level hospital	2021.3	2 weeks

Since the 1980s, with the increasing number of nursing colleges, nursing education in China has been divided into three levels (15): secondary diploma programs, advanced diploma programs, and baccalaureate programs. All three levels of nursing education enroll male students. According to China Health Statistics from 2019, 85.4 % of nurses graduated from 3-year vocational colleges. However, there are few studies on the perspective and experience of clinical practicum of male nursing students in three-year vocational colleges. In fact, there are <20 papers in China Core Collection and China Social Science Citation Index.

The purpose of this study was to explore the perspective and experience of Chinese male nursing students during clinical practicum by using qualitative research methods. Some parts of Chinese culture may help to understand the specific educational background of male nursing. This study may address the needs of male nursing students, facilitate the transition of male nursing students into the role of registered nurses. Furthermore, these results could help promote professional identity of male nursing students and increase the number of registered male nurses within the nursing profession.

## Methods

### Study Design

This study used the phenomenological qualitative study and individual semi-structured interview method to explore the perspective and experience of male nursing students during clinical practicum. The qualitative methodologies proposed by Higgs and Cherry may help develop a better understanding of the complexity in perspective and experience (1).

### Ethical Considerations

Ethical approval was attained from the Institutional Review Committee of Shanghai Tenth People's Hospital. All participants understood the purpose and process of the study. Participants were told that all conversations were confidential. Their participation was voluntary and they can withdraw from the study at any time.

### Participants

A purposeful sample included 20 male nursing students from a 3-year vocational college in Shanghai. The qualification criteria for participation included: (1) completion of all freshman and sophomore courses in all subjects; (2) completion of at least 30 weeks of clinical practicum; (3) volunteered to take part in this study. Interview locations were selected according to students' preferences and available facilities. A total of 20 participants were included in the sample and none dropped out from the study. The detailed characteristics of participants are shown in Table 2.

**Table 2:** Participant characteristics (*n* = 20).

**Characteristic**	***N* (%) or Mean (SD)**
Nursing as the first choice	
Yes	11(55)
No	9(45)
Age	21.35(1.06)
Ethnicity	
Han	19(95)
Hui	1(5)
Place of birth	
Shanghai	8(40)
Zhejiang	5(25)
Anhui	2(10)
Henan	2(10)
Guizhou	1(5)
Jilin	1(5)
Jiangxi	1(5)
Practicum weeks	31.9

### Data Collection

Semi-structured interviews were conducted between March and April 2021. The interview questions in Table 3 were based on previous research (1, 2, 5) and used to explore students' perspective and experience during clinical practicum. The interviews began with basic, less sensitive questions and encouraged interviewees to open up about experience that might influence their willingness to move from nursing students to registered nurses during clinical practicum. If necessary, the interviewer will ask follow-up questions. The interviews were audio recorded, which lasted some 50 min to 1 h and focused on topics linked to male nursing students' backgrounds, their interest in and perception of the nursing profession, and their experience from clinical practicum. The interviews were conducted by two researchers who interviewed participants and two research assistants who took notes. All participants were rewarded with a small present (pen or jotter) for their participation. The data were saturated with dense categories until none of the new information appeared (16).

**Table 3:** The interview guideline: open questions.

1	How and why do you choose a nursing program?
2	Please describe the story of how you joined nursing.
3	How were you interacting with others in the ward as a male nursing student?
4	What advantages or disadvantages have you come across as a male nursing student?
5	What are your career plans for the future?

### Data Analysis

The audio recordings were transcribed verbatim and checked within 24 h after the end of the interview. Data analysis was conducted independently by two experienced researchers using Colaizzi's phenomenological seven-step method (17) (Table 4) to extract themes and sub-themes.

**Table 4:** Colaizzi's seven-step framework for qualitative data analysis.

**Step**	**Data analysis procedure**
1	All interviews were recorded with the audio recording device and then transcribed. Every transcript was read several times and researchers highlighted the important points.
2	Re-read, highlight and extract significant statements directly related to the perspective and experience of male nursing students during their clinical practicum.
3	Formulate meanings from all significant statements. Two researchers checked and reached a consensus.
4	Identify and organize the formulated meanings into theme clusters.
5	Describe the investigated phenomenon exhaustively.
6	The essential structures of the perspective and experience of male nursing students during their clinical practicum were described.
7	Return to the participants for verification.

In the first phase, all interviews were recorded with the audio recording device and then transcribed. Every transcript was read several times and highlighted the important points by two researchers.

In Phase 2, re-read, highlight and extract significant statements directly related to the perspective and experience of male nursing students during their clinical practicum. During this phase, 209 significant statements were extracted and numbered.

In Phase 3, formulate meanings from all significant statements. In this process, the meaning composition was reviewed with PhD with qualitative research experience and graduate students also engaged in qualitative research.

In Phase 4, subthemes and themes are designed based on significant statements. During this process, comparisons are made with the original data to find any inconsistencies. Researchers repeated these processes several times.

In Phase 5, eight subthemes drawn from the statements of the experience were combined to describe thoroughly the perspective and experience of male nursing students during their clinical practicum.

In Phase 6, similar subthemes were organized in larger clusters and three main themes were obtained.

In Phase 7, the participants were informed as to whether the results in Phases 4, 5, and 6 were consistent with their experience. The researchers confirmed that “our perception was correctly interpreted” and “the results were exactly what we wanted to say.”

If disagreements arose, researchers discussed them until reaching a consensus. All participants consented to be contacted once more and supplied their phone numbers to the researchers.

## Results

Participants shared different perspective and experience during their clinical practicum. From Participant's disclosures, three themes were identified: (a) opportunities and challenges brought by gender factors in hospital humanistic environment, (b) gaining experience and growth in clinical practicum, (c) future career planning. Table 5 summarizes this study's results in three main themes, and their sub-themes.

**Table 5:** Main themes and sub-themes categorized from the data.

**Main themes**	**Sub-themes**
Opportunities and challenges brought by gender factors in hospital humanistic environment	Opportunities and challenges for interactions between the participants and nurses Opportunities and challenges for interactions between the participants and patients Opportunities and challenges for interactions between the participants and their clinical instructors
Gaining experience and growth in clinical practicum	Feeling self-worth and professional pride from clinical practicum work Positive perspective and experience of life
The career planning of the future	Continue in nursing profession Withdraw from nursing Not yet been determined

### Opportunities and Challenges Brought by Gender Factors in Hospital Humanistic Environment

#### Opportunities and Challenges for Interactions Between the Participants and Nurses

Most participants felt that female nurses liked male students and gave more support to them than to their female colleagues during clinical practicum. One nursing student talked about men's role as relationship facilitators. “Male students and female students are studying in a clinical environment at the same time every day. Male students always get more attention than females. The nurses more often talk with male students, thus achieving an active atmosphere in the ward. Wow! It's great.“ (Participant 6).

Although male nurses are beginning to be accepted, nursing has long been considered a very feminine profession in China. Use of the term “angel in white” with obvious feminine connotations further perpetuates this stereotype. Previous researchers (18) have shown that terminology used to refer to nursing staff such as “angel,” “nursing” and “sister,” which has obvious feminine connotation further perpetuates feminine stereotype. “Term ‘sister' does not go well with me, and it is difficult for me as a guy to hear other nurses saying that we are a nurse sister team.” (Participant 19).

“These feminine terms create cognitive confusion and depression because we are being laughed at. If we become qualified, for other female nurses, we become a “sister”. I feel that the nurses do not really accommodate male nursing students.” (Participant 17).

#### Opportunities and Challenges for Interactions Between the Participants and Patients

Traditional Chinese culture and Chinese values, which are based on Confucianism, substantially differ from those in Western countries. Sexual ritual is a major issue in Chinese traditional culture. As the Chinese saying goes, people of different gender should not touch each other when passing each other. For this reason, the work of male nursing students in ward care was strongly limited. Almost all male nursing students were forbidden from providing nursing services to female patients. This is especially common in cases, which require taking care of private parts of the body, such as the perineum or breasts. When talking about their experience, some students said that in providing care, the maternal newborn-clinical area was one of the most challenging fields. One student described himself as “feeling as if he was on thin ice, where there were too many things he was not allowed to do…” (Participant 9).

Several other students also described similar feelings. “They argued that it was very inconvenient for male nursing students to care for female patients, who refused them to provide them with care, whereas the male doctors could do it.” (Participant 3).

Meanwhile, some male students expressed that they could provide personal physical care for male patients during clinical practicum. In fact, they pointed out their gender as the advantage. “In some cases, providing intimate care to a group of patients of different genders has created difficulties. Male patients in China sometimes feel extremely nervous and embarrassed because of their gender when they need private care from female nurses. If the nurse of the same gender provides private care, male patients may feel much more relaxed and at ease “(Participant 14).

#### Opportunities and Challenges for Interactions Between the Participants and Their Clinical Instructors

In individual interviews, male nursing students were asked about their experience and perspective on the preferential attitudes of clinical instructors. Most of them did not negate these preferences. They additionally shared the benefits of being male in learning surroundings: they found that male students received more encouragement from their instructors, such as: “My instructor would say that male nurse now have a strong gender advantage, promising job prospects and easing promotion.” (Participant 10).

Male students also discovered that male students were less likely to be punished by their instructors than female students. “I think there are still some differences between men and women, but still, it depends on the instructors' ideas. I find male students more agreeable. During the clinical practicum, clinical instructors gave more coaching and training opportunities to the male students.” (Participant 1).

By contrast, some students shared the unfair treatment by their clinical instructors. One participant recalled being scolded more harshly each time he made a mistake. “I did not inquest my instructor why I was being treated like that. I suppose my instructor thought men have more mental endurance, when in the face of criticism and punishment.” (Participant 7).

The various dynamics are reflected in these narratives, such as supportive, neutral, and opposition dynamics, which require further in-depth analysis of nursing culture.

### Gaining Experience and Growth in Clinical Practicum

#### Feel Self-Worth and Professional Pride From Clinical Practicum Work

Almost all male nursing students gained self-worth and professional identity from practicum work. The majority of respondents said that, in the post-COVID-19 era, working in the hospital as interns was glorious. “My clinical instructor volunteered to go to Wuhan to provide support in 2020. Although he eventually did not get to go because of some personal reasons, he is now participating in epidemic prevention and control in the hospital. I am very proud of him. (Participant 2).

“Now I am studying in the emergency department, cooperating with nurses to carry out rescue work and maintain order. I think my current work is worthy of the cultivation of the country and the college for many years. I have played my role, and I am (also) making contributions to society.” (Participant 15).

“In the hospital ward where I did my practicum, nurses took care of 8 patients per shift. Sometimes, the nurse needed to contact the patients infected with COVID-19 and spend much time taking care of them. Because of the huge pressure, the female nurses were under great physical load. When situations like this happen, men are in a better position due to their natural physical strength.” (Participant 5).

#### Positive Perspective and Experience of Life

There is no denying that male nursing student who enters the clinic for the first time gain a more intuitive feeling of the clinical work than in college. The support and warmth they received from the community and the hospital encouraged them, and they successively thanked people who supported them. “The whole society is admiring and supporting us; the whole country is supporting us. I will study hard and master all the technology to be able to take better care of the patients.” (Participant 4).

“When I was on the night shift with my instructor, relatives of the patient who was lying in bed 10 brought fruit and bubble tea for us and quietly put them in the nurse's office. They left a note saying, ' For the angels who have worked hard!' This made me want to be more kind to everyone in my life.” (Participant 1).

### The Career Planning of the Future

The perspective and experience of clinical practicum affect the determination of Chinese male nursing students to continue or quit the nursing program. Some of the participants announced that the clinical practicum perspective and experience made them reconsider whether they should continue their nursing profession.

#### Continue in the Nursing Profession

Some participants felt that their knowledge in the nursing field was as good as they expected, while it was not as good in other specialties unrelated to nursing. The main factors for staying in nursing were a great sense of accomplishment, the competitive edge related to gender, and job security. Some students said that they planned to pursue nursing careers after graduation. One student was seriously saying, “Because my score from National College Entrance Examination (NCEE) was not enough to gain me entrance to my first school choice, when I went into nursing school, the first year I've really struggled. It was not until my third year of clinical practicum that I really understood the meaning of nursing, which changed my whole view of nursing. Even though the clinical practicum is tiring, I am glad to assist patients in need.” (Participant 8).

“I'll work in a hospital after graduation. Because I am interested in nursing, I will have job security as soon as I graduate from college. I will get a stable job with good growth potential because of the lack of male nurses in China.” (Participant 11).

“I plan to work in the emergency department (ED) or operating room (OR) when I graduate, as I prefer the feeling of operating the machine. I believe that my future is bright because there are more opportunities for career advancement!” (Participant 11).

#### Withdraw From Nursing

Due to traditional cultural and social prejudices, nursing in China is regarded as a low social status occupation. Several students have considered withdrawing from nursing. They often felt anxious about the nursing course content and found that the clinical practicum tasks in hospitals were burdensome. They were uncertain about their prospects for nursing development. One participant stated that, “I would feel very awkward when I stayed in some special wards, such as obstetric or gynecologic wards. If a female student looked at me, even if it was a simulation exercise in perineal care, I would feel very embarrassed. After graduation, I plan to exit from the nursing.” (Participant 12).

According to the traditional Chinese concepts, an income generated by men is the main source of household income. “Salary is vital to me since I have a family to support in the future. Also, in my opinion, nurses' income is not proportional to their hard work. Routine nursing work was boring and hard, and I often felt exhausted.” (Participant 2).

#### Not Yet Been Determined

Some male nursing students have not yet decided whether they should pursue a nursing career after graduation. If male nursing students decide not to become nurses, they need to prepare a new path outside nursing. “I wouldn't say I love nursing, but I should cherish the opportunity to work in the hospital. I don't have to worry about unemployment. As for salary, it's really not high in Shanghai. I plan to buy a house in Shanghai and I expect to be under a lot of pressure to pay the mortgage. (Participant 20).

“I used to wonder if I should go into nursing. At the moment, I haven't fully adapted to nursing work. Maybe I will continue with the advanced level examination and get a bachelor's degree. If I do get a bachelor's degree, I will have many choices for my future job.” (Participant 13).

## Discussion

Most previous studies on nursing have investigated the perspective and experience of male nursing students in clinical practicum using qualitative methods (19–21). Several researchers have reported that male nursing students often feel isolated because they are a minority in a female-dominated profession (22, 23). However, our results revealed the opposite phenomenon that male students had namely very harmonious relationships with their female colleagues. Due to the physical characteristics male nurses have, the acceptance of male nurses in a female-dominated nursing profession has substantially increased. This was supported by a study, which included Japanese male nurses, where the males got respect and recognition from their female colleagues on account of providing help with physically difficult tasks, like lifting overweight patients (24). We found similar results in our research among male nursing students working together with female nursing students. Still, there are not enough similar studies in China, and further research is warranted.

To fill these literature gaps, we conducted this research from unique Chinese cultural perspective, which revealed three unique factors affecting the perspective and experience of male nursing students in China. First of all, being a country with a long history, China has a strong tradition of intrinsic socio-cultural values, which greatly differ from Western countries. On the one hand, in the traditional Chinese concept and culture, nurses are expected to provide a great part of health care services. In contrast, when directly facing the issue of masculinity, it has been found that the concept of masculinity profoundly influences male nursing students (25). The traditional view in China is that men, by nature are not good at nursing. On the other hand, Chinese feudalism gave men a superior position and insisted that male-dominated professions are naturally assumed as higher-status professions (26), such as engineers or civil servants. However, regarding professional rank and educational level, nurses in Asian society are thought to have a lower status compared to other professionals such as doctors. Social identity and gender roles are culturally established (18). Secondly, stereotyping of male nurses and low professional identity may make male nursing students have to confront bias and misunderstandings from their family and friends. The social ideology seems to make it almost impossible for Chinese men to become a nurse and do nursing work (2). Some male nursing students mentioned that they get mixed feelings from their parents for their choice of the nursing profession and aggressive humor remarks from their friends, such as teasing, ridiculing, and mockery. Finally, the Chinese are reluctant to convey their emotional and mental distress (27), especially Chinese men. Most male nursing students who were unwilling to express their perspective and experience during their clinical practicum could not receive support from others as a means of alleviating distress. Since male nursing students cannot avoid stressors, they need to deal with stressors. If male students are unable to express and manage their stressors, they can influence their achievements as nurses and mental health, mind, and occupational satisfaction (28). Thus, it can be concluded that the concerns listed above have hindered the development of male nursing students in China.

However, not all male nursing students have a negative view of their career development. On the one hand, as nursing requires physical strength and high technical skills, many male nursing students believed they have an advantage over girls in clinical settings such as intensive care units, operating rooms and emergency departments (2). On the other hand, clinical practicum is the primary period when the formation of nurses' professional identity (6). As the students mentioned in the interview, most of male nursing students began to truly understand the significance of nursing work after entering clinical practicum. The goal of the nursing profession is the same as that of the medical profession: to provide each patient with safe, humane and the latest personalized care (29). In particular, since the outbreak of COVID-19, many nurses in China have volunteered to participate in the front-line fight against the virus. This behavior further changed male nursing students' professional identity of nursing. It is necessary that male nursing students be supported in preparation for and during practicum to reduce their adverse clinical experience and amplify positive feedback (30).

Male nursing students are still in the minority as potential future nurses (1). Within traditional Chinese culture, male nursing students are not willing to express their psychological pressure and mental distress during their clinical practicum, which is a barrier to switching roles of RN in their careers. A professional alliance assists and accompanies male nursing students for all essential activities of daily working. In 2014, The Northern Prairie Alliance (NPA) was created as a section of the American Association for Men in Nursing (AAMN) at Northern Illinois University to create a supportive background for male nurses (American Association for Men in Nursing (AAMN), 2018). As a public voice for encouraging male nursing at the country level, AAMN membership is open to any nurse, regardless of gender, in order to better promote male nursing (AAMN, 2018). Likewise, the NPA welcomes every gender student. Therefore, we wish to establish a male nursing Student Union in China, which should be public to all male and female nursing students, to fulfill their need for gender equality and diversity. In addition, nursing managers in China should adopt measures designed to help male nursing students relieve peer pressure and promote professional identity actions through male nursing student unions, such as psychological consultation, professional lectures, or public welfare activities.

As more people realize the advantages men can have in nursing, such as physical strength, technical ability, calmness, and autonomy, hospitals may be more willing to recruit male nurses (31). Male nurses are competitive in the job market and are regarded as a scarce resource by hospitals (22). On the one hand, male nurses have relative advantages in physical strength, energy, and emergency ability. Male nurses are more able to handle heavy work and night shifts (5). They are also more sought in emergency departments, intensive care units, and other critical care departments. For example, male nurses have more obvious physical advantages in the treatment of severe COVID-19 patients. They can carry critically ill patients and turn over paralyzed patients. In the face of mass casualties, male nurses have more obvious advantages in certain areas, such as cardiopulmonary resuscitation, which requires greater physical strength. Male nurses also had important roles in transporting patients and providing first aid for patients from the Wenchuan earthquake in 2008. On the other hand, men and women tend to have different thinking styles. The original nursing thinking mode has greatly changed, as well nursing working mode and habit of the nursing group. At the same time, diversified thinking patterns make nursing work more dynamic and innovative. Yet, stereotyping of male nurses in China has led to considerable prejudice against male nursing students, leading to stigmatization and ridicule. One means for implementing the shift is the public and mass media in China that can increase the positive image of male nurses. E.g., a recruiting poster at the Oregon Center for Nursing in 2003 had a slogan, “Are you man enough to be a nurse?” emphasizing that male nurses were manly and that nursing was an occupation that can only be done by'real' men” (1) The previous research has shown that the mass media have an explicit influence on the social perspective of nursing as a female profession (32). In countries such as Australia and Canada, media positive description of male nurses has been associated with the steady rise of a number of men entering the nursing profession (33).

Nursing has long been thought of as a feminine occupation in Chinese society as it is associated with the image of Chinese women being responsive and nurturing (34). High-quality Nursing Service in China places too much emphasis on home care and private care. Cleaning, bathing, nursing, and other work add to the burden of nurses (2) and are in contradiction with the stereotypical view of masculinity advocated by traditional Chinese culture. In Chinese culture, rebuilding current gender norms is critical to eliminate biases and change the definition of masculinity, which in turn contributes to the increasing number of registered male nurses. The Chinese nursing development plan (2016–2020), which the Chinese National Healthcare Commission issued, emphasizes the significant importance of technology in nursing clinical education (13). Greater focus on the therapeutic rather than care aspects of nursing may also increase the appeal of male nursing students in China toward this profession (2). Male nursing students apparently get a sense of achievement and superiority when they are engaged in the technological aspects of nursing work. Consequently, to reduce the loss of male nurses, the education department should actively promote skills training (33). For instance, some students suggested that nursing schools and nursing departments should train male nursing students for professional positions, taking into account their strengths and personalities (35). American Association of Colleges of Nursing (AACN) believes that communication ability is one of the four core competencies of nurses (36). It is very important to carry out systematic special training of clinical communication ability for nursing students in college. For example, educators can introduce communication cases in the course and encourage male nursing students to participate in discussions and role play.

Furthermore, men are traditionally expected to be the breadwinners of the Chinese household. The salary of Chinese nurses is not enough to support a family in fact. Low income may contribute to the lower attraction for male nursing students to pursue the nursing profession (37). A previous study found that men in Macau did not think a lot about leaving nursing, while male nurses on the mainland often felt uncertain about their career prospects. At present, the salary of nurses in Macao is much higher than the average income levels (38). Moreover, in most Hospitals in China, nurses with diplomas and degrees have similar responsibilities and salaries, with a difference of about $50 (6), which may influence male nursing students' professional recognition and ambition. Chinese lawmakers with a nursing background have been calling for better social welfare for nurses.

In order to facilitate the male nursing students to complete the role transform, the practicum hospital should improve the teaching management structure. Establish an expert teaching management committee to make a three-level management framework of director-nurse manager-clinical instructors. The director is responsible for the overall control of the design, implementation and quality of training for male nursing students. Nurse manager coordinate targeted teaching and training content. Clinical instructors carry out specific teaching and training combine the characteristics of male nursing students (39). At the same time, clinical instructors must receive effective clinical nursing education training and provide constructive feedback to male nursing students (40). In addition, A previous study (12) has indicated that attention should be paid to the harmonious tutor-student relationship in clinical teaching. The harmonious tutor-student relationship can help male nursing students to enhance nursing skills and become compatible with the clinical environment faster. Finally, strategies are needed to refocus work in the field of male nursing students and encourage more male nurses to act as instructors, educators, and role models (41).

## Conclusion

More and more Chinese male students take the initiative to participate in the nursing profession; however, the traditional cultural norms and deep-rooted social values in China hinder men from pursuing nursing as their career. Researching the male nursing students' perspective and experience during their clinical practicum can provide implications for future nursing education. Additionally, as suggested in this study, different kinds of support should be provided for the development of male nursing students, including those by social unions, mass media, government policies, hospitals, and colleges. These results can help better address male nursing students' needs and complete the role transform of registered nurses. It can also promote their professional identity and make these students stay in nursing positions.

## Data Availability Statement

The original contributions presented in the study are included in the article/supplementary material, further inquiries can be directed to the corresponding authors.

## Author Contributions

JJ and YL: study design. JJ, YL, PH, CY, and XD: data collection, data analysis, and manuscript preparation. All authors contributed to the article and approved the submitted version.

## Funding

This work was supported by Medical Educational Reform Project of Tongji University, 2021YXSZ01. The funding sources provided financial support for the conducts of the research.

## Conflict of Interest

The authors declare that the research was conducted in the absence of any commercial or financial relationships that could be construed as a potential conflict of interest.

## Publisher's Note

All claims expressed in this article are solely those of the authors and do not necessarily represent those of their affiliated organizations, or those of the publisher, the editors and the reviewers. Any product that may be evaluated in this article, or claim that may be made by its manufacturer, is not guaranteed or endorsed by the publisher.
